# Notes and News

**Published:** 1898-04-02

**Authors:** 


### Notes and News.

(Collected and Communicated.)

The Worshipful Company of Skinners have granted a sum of £1,000 towards opening the closed wards of the City Orthopædic Hospital.

Mr. J. J. Colman has purchased from the governors of the Jenny Lind Infirmary the site on which the old hospital stands, and he is generously converting this into a model playground for the children of Norwich, where open spaces are by no means plentiful. Steps are now being taken to begin the building of the new Jenny Lind Infirmary, which is to contain 40 beds. Until the whole of the money needed is subscribed, it is proposed to put up only a portion of the hospital, for which purpose about £8,500 is required, and is in hand.

The death-rate from measles continues high, 143 deaths from that cause having occurred in London last week, or 88 above the average. In some provincial towns, however, this disease is even more fatal, being 2·8 per 1,000 in Oldham, 3·8 in Bristol, and 4·0 in Leicester, against 1·7 in London. It is interesting to note how measles is supplanting scarlet fever in public importance. In none of the large towns did the death-rate from scarlet fever reach 1·0 per 1,000; and while in London 143 persons died during the week from measles, the deaths from scarlet fever in the same period amounted only to 15.

The air continues to be thick with reports of "scandals" in relation to Metropolitan Asylums Board affairs, and the matter of Mr. Brown and the bricks has given much fine opportunity for virtuous writing in the Radical papers. At the meeting of the managers at which the whole matter was discussed it was maintained that the attack on Mr. Brown was a "dishonest" one, and that his bricks having been bought in the open market he could have no control over such transactions. The way in which the cost of the Brook Hospital was allowed to exceed the estimates to the tune of about £50,000 is another matter, and we cannot but suppose that, perhaps in consequence of the architects having been previously employed by the Board in emergency work, in the doing of which they were practically given *carte blanche*, a certain laxity in regard to formalities had grown up in the relationship between architect and employer, which undoubtedly ought not to be allowed in the execution of great public works.

Lord Lister will preside at the annual festival dinner in aid of King's College Hospital, which is to be held on May 3rd at the Hotel Metropole.

A festival dinner in aid of the Hampstead Hospital at Parliament Hill will be held at the Grand Hotel on Wednesday, 27th April. Mr. T. Sansome Preston will be in the chair.

The Bishop of London has consented to preside at the festival dinner in aid of the Hospital for Sick Children, Great Ormond Street, to be held on Thursday, April 28th, at the Hotel Metropole.

Lord Rothschild, the president of the Royal Hospital for Diseases of the Chest, City Road, has given 200 guineas to the Festival Dinner Fund, to which also Messrs. N. M. de Rothschild have contributed 100 guineas.

The Boscombe Hospital is to be enlarged and improved. A new site is to be purchased, and a strong effort made to collect the necessary funds for the new building. The present hospital accommodates only ten patients, and this is found totally inadequate for the population. Incidentally, it was pointed out at the annual meeting of subscribers that the Armenian Relief and the Indian Famine Funds had materially lessened local subscriptions to the hospital.

It is gratifying to note that the teaching of First Aid for sailors is one of the most appreciated works done at the Missions to Seamen's Institute at Poplar. The complete isolation of the sailor at sea renders it imperative that knowledge of what to do in case of accident and emergency should be widespread among nautical people. A case is recorded of an able-bodied seaman on a sailing ship meeting with a severe accident to his head. The first mate sat for upwards of an hour on the deck holding the profusely-bleeding head on his knee, and appealing to all the crew, "Didn't anybody know how the bleeding could be stopped?" Had it been a question on astronomy or navigation, a dozen answers would have been ready; but on a matter of first aid all were dumb. Not a man of a large ship's crew had an idea of "what to do."

The death of a Swedish emigrant, in a Liverpool boarding-house, from poisoning by carbonic oxide gas gives prominence afresh to very important questions as to the propriety of gas producers substituting such a virulently poisonous compound as water gas for the ordinary coal gas to which the public is accustomed, and for the supply of which the gas companies ostensibly obtained their Acts of Parliament. It is well known that most of the "stuffiness" of atmosphere in many dwelling houses that is commonly put down to defective drains is really due to leaky gas pipes, and it is generally recognised that an escape such as will produce this drainy odour, although it will spoil appetite, produce sore throat, and generally deteriorate the health, is not immediately dangerous to life. The whole aspect of the matter is immediately changed, however, when water gas is suddenly substituted, or used to any large degree as an adulterant "to meet an emergency." A small leak, which under ordinary circumstances, does no harm, may then become a source of immediate peril to life.

